# Mitochondrial damage revealed by immunoselection for ALS-linked misfolded SOD1

**DOI:** 10.1186/1750-1326-8-S1-P70

**Published:** 2013-10-04

**Authors:** Sarah Pickles, Laurie Destroismaisons, Sarah Peyrard, Sarah Cadot, Guy Rouleau, Robert Brown, Jean-Pierre Julien, Nathalie Arbour, Christine Vande Velde

**Affiliations:** 1 Centre de recherche du CHUM, Universite de Montreal, Montreal, QC, Canada; 2 Department of Biochemistry, Universite de Montreal, Montreal, QC, Canada; 3 Department of Medicine, Montreal, QC, Canada; 4 Department of Neurology, University of Massachusetts Medical School, Worcester, MA, USA; 5 Department of Psychiatry and Neuroscience, Université Laval,, Laval, QC, Canada

## Background

Amyotrophic lateral sclerosis (ALS) is a late onset neurodegenerative disorder characterized by the loss of motor neurons resulting in a progressive paralysis that ultimately leads to death. Although the majority of cases are sporadic, some cases are caused by genetic mutation in Superoxide Dismutase 1 (SOD1) where mutations yield misfolded protein leading to an unknown toxic gain of function. Mitochondria have long been considered a target of mutant SOD1 toxicity. Mitochondrial morphological abnormalities are an early feature in the pathology of ALS in both patients and animal models and various aspects of mitochondrial dysfunction have been reported in ALS models although no consensus exists. How this primarily cytosolic protein can affect mitochondria remains to be established. Using antibodies specific for misfolded SOD1, we have previously shown misfolded SOD1 to be associated with the outer membrane of spinal cord mitochondria from ALS animal models. We hypothesize that the mitochondrial association of misfolded SOD1 negatively impacts key aspects of mitochondrial function.

## Materials and methods

Using a novel flow cytometric assay, spinal cord mitochondria isolated from rodent models overexpressing mutant SOD1 were immunolabelled with an antibody targeted to misfolded SOD1 (B8H10), and fluorescent indicator probes were used to assess damage tracking with the presence of the misfolded protein on the mitochondrial surface.

## Results

Using two antibodies targeted to misfolded SOD1, B8H10 and C4F6, we find selectivity for the B8H10-reactive misfolded SOD1 for spinal cord mitochondria isolated from SOD1^G93A^ rats. In contrast, C4F6-reactive SOD1 is not mitochondrially associated. Here, we report an age-dependent deposition of B8H10-reactive SOD1 on spinal cord mitochondria in two different mutant SOD1 models. The presence of misfolded SOD1 on the mitochondrial surface appeared before other markers of pathology including weight loss, gliosis and significant loss of motor neurons. Mitochondria labelling positively for B8H10-reactive SOD1 had increased mitochondrial volume, produced excess superoxide, and had increased exposure of the toxic BH3 domain of Bcl-2. Lastly, B8H10-reactive misfolded SOD1 was found in lysates and mitochondrial fractions of lymphoblasts derived from ALS patients carrying SOD1 mutations, but not in controls.

## Conclusions

This assay identified dysfunction of a mitochondrial subpopulation coated with B8H10-reactive SOD1 which appeared prior to classic markers of pathology, and thus may be especially relevant to disease onset. That B8H10-reactive misfolded was also present in patient lymphoblasts as well as animal models demonstrates the universal nature of this misfolded conformer. How misfolded evokes these damages to mitochondria is presently under evaluation.

